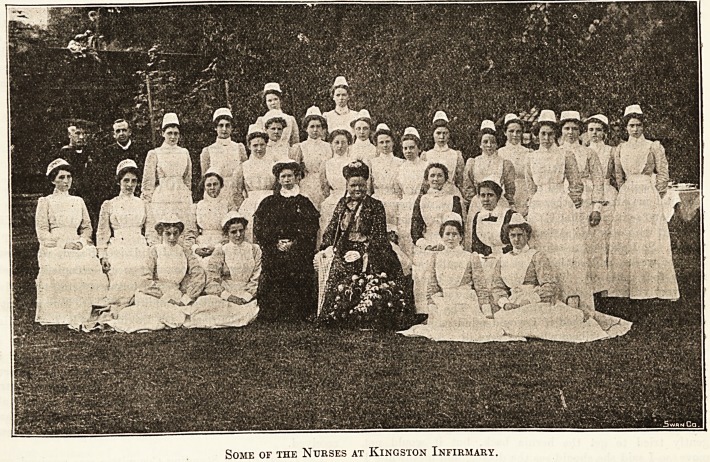# The Hospital. Nursing Section

**Published:** 1905-12-23

**Authors:** 


// The Hospital.

Nursing Section.

Contributions for "THE HOSPITAL," should be addressed to the EDITOR, "THE HOSPITAL" NURSING SECTION, 28 & 29 Southampton Street, Strand, London, W.C.

No. 1,004.—VOL. XXXIX. SATURDAY, DECEMBER 23, 1905.

### Notes on News from the Nursing World.

### OUR CHRISTMAS CLOTHING DISTRIBUTION.

ON Tuesday last all the gifts for our Christmas distribution of clothing were on view at the offices of THE HOSPITAL, and later on in the same afternoon were packed up and despatched to various hospitals. The cold snap in October must have set many kind and clever fingers knitting and crocheting, for the stock of woollies was a large one. The appeal which we made in our columns last year was certainly responded to by our kind readers, for the total of garments was quite above the average. The number of petticoats contributed was a little disproportionate. There were fewer women's and children's nightdresses this year, which is a pity, as these are such indispensable garments. The noteworthy contributions included a really delightful parcel—beautifully knitted vests and shawls, and a dozen gaily dressed dolls and two paint-boxes; a number of very dainty garments, including two nightdresses and two petticoats; a parcel of very warm men's shirts, also women's stockings and nightdresses; a pile of socks and stockings; and a batch of warm, knitted vests and petticoats. The Editor of THE HOSPITAL contributed some warm men's clothing for which there was much need, but except for stockings there were very few articles for boys. The babies, as usual, came in for a good deal of attention, the tiny garments being so fascinating to make. We have still to acknowledge contributions from Miss Catmur, through Nurse Fenckenstadt; Miss Francis; Policy No. 4,219; A. L. K., Cheltenham; Miss L. J. Atkin, Torquay; Nurse Lane, Aylesbury; Miss Woodhouse, Teddington; Mrs. Gage, Brockley; Policy No. 545; and a lady whose name is not given.

### A CHRISTMAS NUMBER WRITTEN BY NURSES.

IT is intended to make the feature of our Christmas Number of 1906 a series of incidents occurring at the Christmas season this year, and we therefore invite nurses engaged in general or special hospitals, in Poor-law infirmaries, in mental institutions, and in district or private work, whether at home, in the Colonies, or abroad, to send in contributions, varying from 500 to 1,000 words, not later than March 31 next. Original photographs illustrating the articles will be very acceptable, and these and the literary contributions used will, of course, be paid for at the usual rate. Preference will naturally be given to the most carefully and brightly-written accounts, but incidents of exceptional interest will not be passed over because the writer is deficient in literary style and composition. The contributions should in all cases be addressed to the Editor of the Nursing Section of THE HOSPITAL, and marked outside "Christmas Incidents."

### THE DURHAM ASYLUM SCANDAL.

IN publishing last week a remarkable letter from the matron of the Durham County Asylum, containing serious allegations of neglect against the stores officials, and of injustice on the part of the Committee, we considered it necessary to invite the county authorities to explain. The invitation has not, so far, met with any response. We have, however, received private letters from medical men and others in the district commenting on the case, in one of which the writer says, "Such gross injustice as that perpetrated in the case of the matron and Dr. Hunter has seldom, I hope, occurred in the Asylum world." It will be seen, in another column, that Dr. Hunter writes to express his sympathy for the matron. The probationers of the Durham County Asylum also write us collectively, thanking us for inserting the matron's letter. They confirm the statement that the patients suffered owing to the inability of the stores officials to supply the requisitioned articles mentioned, and express their great grief at losing one of their medical officers and the matron, "because they supported the right," adding that "they had our interests and those of the patients at heart, and although we were not encouraged, we upheld their action, and we shall never forget the unjust treatment they have received." We are informed that the editor of the local paper refused to insert the matron's letter which was sent to us on the ground that he makes it "a rule not to interfere with public bodies and their officials." As, on the contrary, we hold that it is desirable that the actions of public bodies supported by public money should not be concealed when principles of vital consequence are at stake, we gladly afforded Miss Mitchell the hospitality of our columns. They are, of course, still open to any of the authorities of Durham County Asylum who may recognise that silence maintained justifies damaging conclusions.

### NURSING TOPICS AND PARTY POLITICS.

THE people who are anxious, for purposes of their own, to curry favour with politicians are very angry because we have urged upon the officials of nursing organisations the duty of doing their utmost during the General Election to keep nursing topics outside the pale of party politics. It is a matter of no concern to us whether or not the Women's Liberal Unionist Association, or any other body of party politicians continue "to keep in touch with the progress of the State Registration movement." They must, of course, do as they please, and we do not presume to imagine that any attitude of mind on our part will affect their action. It is solely for the sake of preventing nursing topics from being dragged through the mud of party politics that we demur to meetings such as the one on which we lately commented; and we are glad to know that this view of the matter is widely shared in the nursing world, apart, of course, from those restless spirits whose thirst for notoriety blinds them to the best interests of the cause they profess themselves eager to serve.

### SPOTTED FEVER AT FINCHAM.

We understand that the accounts of the epidemic of spotted fever at Fincham, near King's Lynn, which have appeared in the daily press are somewhat exaggerated. The facts are these:—The number of cases has not exceeded fourteen, two deaths have occurred, the patients are being nursed and isolated as far as possible, but there is no sort of panic, and the village itself is not isolated. Dr. Farrar, one of the Local Government Board Inspectors, is inquiring into the causes of the outbreak. No nurses whatever have been sent from King's Lynn to attend the patients.

### PROBATIONERS AND MEAL TIME.

We are glad to learn that special precautions are taken by the matron of the Poor-law Infirmary at Kingston-on-Thames in respect to the health of the probationers under her care. Miss Smith, in the interview with our Commissioner, which appears in another column, states that she does not allow them to be absent from meals or to go on duty before they have had breakfast. She includes meals in the duty time. This is an excellent arrangement. As the matron of Kingston Infirmary says, it prevents any hurrying over meals in order to catch trains, which, under other conditions, is by no means infrequent. It is a drawback that the nurses' quarters are not all under one roof at Kingston, but this will doubtless be remedied before long, and, generally speaking, the provision for the staff is all that could be desired. The illustration we give is from a photograph taken during the visit of the Duchess of Albany to the Infirmary, but it does not include the matron nor the ward sisters.

### THE DEAD AND THE LIVING HAND.

A fortnight ago we announced that the sum of £10,400 had been left by the late Mr. Noble for the maintenance of five district nurses in the Isle of Man. This week we have to record the fact that Captain Partington has presented to the Borough of Glossop as a Christmas box a sum of £30,000 for the erection and endowment of a nursing and convalescent home for the sick poor of the town and for the provision of trained nurses to visit the sick poor at their own residences, and instruct mothers in hygiene, housekeeping, and the rearing of children. We heartily congratulate Glossop upon the public spirit of a popular townsman, and Captain Partington upon the admirable purpose to which he has devoted such a handsome sum while he may hope to have the gratification of seeing his useful and much-needed project completed.

### A QUESTION OF REMOVAL.

The decision of the Committee of the District Nurses' Home at Ardwick, Manchester, to remove from Ardwick Green to Plymouth Grove has given rise to complaints that the nurses will not be so readily available in some of the districts where they are most wanted. But a very reasonable explanation of the change has been made on behalf of the Committee. There is no question of the urgent need of increased accommodation, and the idea of rebuilding on the present site was at first considered. It was rejected because it would have involved two removals; because it would have cost from £4,000 to £5,000, as against the £1,500 asked for now; and because the surroundings at Ardwick Green are rapidly degenerating. As, in addition to these considerations, the matron is confident that the nurses can carry on their work as well from Plymouth Grove as from Ardwick Green without detriment to the interests of the patients, we think that, though a removal from one locality to another inevitably causes some inconvenience and should be avoided when practicable, the action of the Committee in this instance cannot be condemned.

### QUEEN ALEXANDRA'S MILITARY NURSING SERVICE.

We are officially informed that Miss J. G. Dalton, Miss E. B. Darnell, and Miss M. German have been appointed staff nurses in Queen Alexandra's Imperial Military Nursing Service. Miss J. S. G. Gardner (staff nurse) has been posted to the Military Hospital, Chatham, and Miss M. S. Williams (staff nurse) to the Military Hospital, Colchester. Miss A. Guthrie (sister) has been transferred from Pretoria to Middelburg, Cape Colony, and Miss L. M. Todd (sister) from Maritzburg to Middelburg, Cape Colony. Miss M. E. Harding, Miss J. Hoadley, R.R.C., Miss E. J. Martin, Miss A. Nixon, and Miss S. I. Snowdon have been ordered to England on expiration of tour abroad.

### LADY ABERDEEN AND DISTRICT NURSING IN IRELAND.

The Countess of Mayo has been elected Vice-President of the Committee of the District Nursing Fund in Ireland, founded by the Countess of Dudley while her husband was Lord Lieutenant. We conclude it is assumed that the Countess of Aberdeen, wife of Lord Dudley's successor, will take the place of Lady Dudley, who was President and Treasurer. Lady Aberdeen has always manifested a keen interest in district nursing, and we do not doubt that she will spare no exertion in order to maintain and extend the usefulness of the work which Lady Dudley so successfully inaugurated.

### JUNIUS S. MORGAN BENEVOLENT FUND.

We have received an anonymous contribution of £1 for the Junius S. Morgan Benevolent Fund from Edinburgh, which has been sent to the Secretary, who desires us to express grateful thanks to the donor.

### PENSIONS FOR SCOTTISH CO-OPERATION NURSES

At the annual meeting of the Glasgow and West of Scotland Co-operation of Trained Nurses, Professor Clark, in moving the adoption of the report, observed that the institution had made extraordinary progress. This is true enough. The number of cases nursed in the year was 1,772, and the earnings amounted to £10,533—a big record, seeing that the Association has only been established thirteen years. It is curious, however, that, as compared with the previous year, the gross earnings of the nurses, of whom there are now 181 on the roll, showed a decrease of £191. At the conclusion of his speech Professor Clark emphasised the necessity of making provision for the nurses in their later days, and commended the example of the Victoria Infirmary, where they had agreed not only to encourage nurses to take out policies in the Pension Fund, but had agreed to pay half the premiums. Ultimately, Dr. David Newman intimated that he hoped to bring forward a proposal at the next meeting of the Executive.

### TAUNTON BRANCH OF THE SOCIAL UNION.

On Thursday and Saturday last week Dr. Joscelyne gave demonstrations of the machinery and working of the Röntgen rays, high-frequency battery, and other electrical apparatus, at his residence in Taunton. The meetings were attended by a number of nurses from the surrounding neighbourhood, belonging to the Taunton branch of the Nurses' Social Union, who greatly appreciated Dr. and Mrs. Joscelyne's kindness and hospitality.

### A WOMAN WITH THE NURSING VOCATION.

Devonport has lately lost one of its most respected inhabitants. We refer to Mary Frances Xavier, who, for thirty-five years, was Mother Superior of the Society of the Holy Trinity in the town. As far back as 1872, when Devonport was visited by an epidemic of small-pox, she received and tended the sick in a building prepared for the purpose in Ascot Priory grounds. Subsequently, she had charge of the Permanent Ward for Convalescents and Incurables. Here her ministrations were greatly appreciated, because of her genial and kindly manner, and the patients valued her friendship as well as her care. During the last seven years of her life she was herself a great sufferer.

### VILLAGE NURSES IN LINCOLNSHIRE.

A generous offer was mentioned by Lord Yarborough, who presided at the annual meeting of the Lincolnshire Nursing Association, Edith, Countess of Winchilsea, having on account of ill-health resigned the office of president. At present the income of the organisation is only a little over £200 a year and the Committee are anxious to increase it to £500. Lord Yarborough stated that a gentleman, who did not wish his name to be disclosed, had written to Lady Thorold saying that he was willing to guarantee £100 a year for five years, if the county would find the other £400. This gratifying intimation, which the chairman naturally used in order to enforce his appeal for more help, elicited from Sir George Doughty, M.P., the information that in the village of Waltham it cost the residents £45 to £50 a year for their nurse, but that they were never without money, and had an endowment fund of £160. His argument was that if that could be done in a comparatively poor village, it could be done in every district in Lincolnshire. The village nurses are not, of course, fully trained, but they are under the supervision of the county superintendent.

### THE DANGERS OF METHYLATED SPIRIT.

On Friday a coroner's jury were engaged in inquiring into the circumstances of a fatal accident to a foundling at the Fulwood Workhouse, Preston. The child, who was suffering from measles and bronchitis, was placed in a cot with a canopy, into which steam was admitted from a kettle heated by methylated spirit. This was standing on a chair by the bedside. While the nurse was at tea, the child's clothing by some means caught fire, and though the patient was quickly stripped, he soon died from the injuries received. The jury, in returning a verdict of accidental death, recommended that such apparatus as the kettle should have a more solid basis than the chair. But the fact is that methylated spirit is altogether too dangerous to employ for heating purposes in cases of this kind, unless there is someone always in attendance.

### SUCCESSFUL ACTION BY A NURSE.

At Brompton County Court on Tuesday a case was heard in which the plaintiff was Miss Carey, a trained nurse, who claimed £41 7s. 8d. for professional services to Mrs. Fraser, the wife of a former Captain in the Army. For the defence it was urged that Mrs. Fraser would be ruined if she were made responsible for her husband's debts; but as it was proved that she herself engaged the nurse, and possesses a considerable private income, judgment was given for the plaintiff with costs. We regret that a lady who, in cross-examination, admitted that she enjoys an income of about £400 a year should have endeavoured to avoid the payment of fees due for nursing, and we are glad that Miss Carey has obtained the judgment to which she was so clearly entitled.

### A START AT SALTASH.

The promoters of the Saltash Nursing Association having received promises of subscriptions amounting to £90, it has just been decided to form a permanent Committee for the ensuing year. A discussion took place at the meeting concerning the qualifications to be held by the individual it is proposed to obtain, and eventually it was determined not to engage a Queen's nurse, but a hospital-trained nurse, who would be acceptable to the Cornwall County Nursing Association.

### SHORT ITEMS.

The number of nurses employed on the district staff of the Manchester and Salford Sick Poor and Private Nursing Institution is not eighty-four, but sixty-four.—We are asked to state that the illustration in our issue of December 9, described as "The Church at West Malling Mental Asylum," represented a picture of the interior of the parish church.

### The Nursing Outlook.

"From magnanimity, all fear above;
From nobler recompense, above applause,
Which owes to man's short outlook all its charm."

### A HAPPY CHRISTMAS.

Our present issue will reach the hands of many of our readers on the eve of Christmas. We have had twenty years' pleasant association with them, and time welds together the sympathies of editor and readers, and creates a bond of sympathy and good feeling which increases as the years roll by. We are therefore in the proud position of addressing friends, for every reader of The Hospital may be so regarded, and in the truest spirit of friendship we wish each and all a happy Christmas and a prosperous New Year. Happy, in the consciousness of good work for others done in the right spirit and in the best way. Prosperous, in the sense that the mere act of doing has raised the character of the individual, has extended the range of his or her vision, and sweetened the atmosphere of their daily life, so that every sense and feeling has been quickened and purified to the unselfish ennoblement of the worker.

We have often treated of hospitals as exercising a humane influence on the public at large. To-day we will consider the humane influence which the hospitals exercise upon everybody, from the physician to the patient, who becomes intimately associated with their work. Christmas Day, when the majority of people are enjoying themselves at home, is an occasion when some hospital should be visited by every thoughtful man or woman, who takes an intelligent interest in the social institutions of his day and generation. A visit to a hospital on Christmas Day must raise the spirits of the visitor, and impress upon all, who follow the course here suggested, the extent of the influence which hospitals do, in fact, exercise for good, upon tens of thousands of people in this country, especially.

One charm the visitor cannot fail to experience is the contrast between the dreary and deserted streets outside, and the activity, brightness and sociability to be found inside hospitals, on Christmas day. Entering a large ward the visitor is at once struck with the beauty of the decorations, and the evidence of the loving care for the patients, especially for the children, which is everywhere forthcoming. These characteristics are not confined to one ward but are common to all. If the hospital has the advantage of being attached to a medical school, there will be found the most brilliant decorations, accompanied by every charm which the energy of youth and the best instincts of early manhood can produce, to cheer the patients and make them happy and forgetful of their sufferings. The outside world little realises the excellence and beauties which the combined efforts of students, nurses and medical staff have introduced, into hospital wards, nowadays, at Christmas time. It is not too much to claim that, apart altogether from the service rendered to the community by the alleviation of suffering and the cure of disease, the modern hospital, by its discipline and methods, thanks largely to the presence of educated women nurses, inculcates in the patients a spirit of unselfishness and consideration for others, which cannot fail to make them doubly welcome, when they leave the hospital and return to their homes and families. A child, accustomed to the rough usage of streets and slums, is often fretful and fractious on admission, but the quick intelligence which the struggle for existence develops in city children causes them to recognise the tender thoughtfulness of both doctors and nurses, and so gives them confidence which secures their co-operation during treatment, especially where the dressing of wounds may cause pain to the patient. The atmosphere of a modern hospital, in a great city, is very often a new and a better atmosphere for the majority of the patients. Its effects have a direct influence for good upon the morale and character of every one who may come under this influence.

A hospital is a small republic in its way. The sister of each ward, with her own staff, are zealous for its reputation. The results to be seen in the wards of Guy's Hospital, especially, at Christmas time, are well worthy of the attention and inspection of artists generally. The infinite variety of design, not only in the character but in the detail of the decorations, is remarkable. In the manufacture of ropes of evergreen alone a visitor may sometimes see more than a dozen separate designs, some of them fanciful, most of them effective, and all surprisingly beautiful. Most wards too contain a Christmas tree, and the beauty of the flowers, with the bright and attractive decorations of the tea tables, where every one is welcome, are often remarkable. Small wonder, then, if few family gatherings produce more happiness, or exercise a wider influence for good, than the gatherings in the hospital wards on Christmas day. Here everybody is so friendly and pleasant that all must feel at home and at peace.

We hope that those of our readers, who are not connected with hospitals, will make up their minds to visit one hospital, at least, on Christmas day 1905, when we promise them a genuine surprise, real pleasure, and many novel sensations. It should excite a generous interest amongst the healthy of all classes, who have it in their power to give prompt expression to their gratitude, in cash. This is an age of novelty. So we recommend these visits to hospitals on Christmas Day to all who value the privilege of life, as opposed to mere existence, and of pleasure in contradistinction to killing time.

### Abdominal Surgery.

By Harold Burrows, M.B., F.R.C.S., Assistant Surgeon to the Bolingbroke Hospital.

### SYMPTOMS IN ABDOMINAL DISEASE. SECONDARY SHOCK.

(*Continued from Page* 146.)

### Value of Warmth.

Foremost of all the means of lessening shock is the proper supply of warmth to the patient. It must be remembered that when an individual is in a condition of shock the processes of metabolism are practically arrested, and so the normal amount of heat is not being produced in his tissues. Added to this is the circumstance that during an abdominal operation a considerable area of skin is laid bare, and in this way heat is rapidly lost. The loss of heat is not only harmful in itself, but it greatly accentuates shock.

Before a patient is taken to the operating-theatre care should be exercised in order that he is warm to start with, and during his absence in the theatre the bed should be well warmed for his return. It is extremely bad practice to place a patient who has just undergone a serious operation into a cold bed.

But the chief concern should be the prevention of coldness during the operation. The temperature of the operation-room itself should be above 80° F. If this cannot be achieved the nearest approach possible to that degree should be secured. Where an operation is to be performed in a private house the fire should be lighted over night in the room selected for the operation, as a rule. Modern operating-tables commonly have some arrangement for keeping the patient warm, but where no such provision is made, a large water-pillow, filled with hot water, should be placed on the table under the blanket on which the patient lies. In very severe cases, and in circumstances which do not permit of a properly heated operation-room, and always in the case of babies, the limbs should be swathed in wool and bandaged. Another important point, and the one which has the greatest chance of being overlooked, is the constant supply of warm lotion for the surgeon and his assistants. As soon as the contents of a lotion-bowl have become cooled they should be replaced, for no lotion below 100° should be allowed entry to an abdominal wound, or, in fact, any other wound, during an operation. Below is a chart showing the result of negligence in such matters. The patient underwent a moderately severe operation. Skilled nursing assistance was not available, with the result that several of the precautions mentioned above were neglected—the lotions were cold, the patient was returned to a cold bed and no proper steps were taken to keep her warm during the operation. She nearly died of cold in consequence, although the operation took place on a warm summer day in London. So far, the treatment described has been preventive, including the proper preparation of the patient and the proper precautions during the progress of the operation. When the operation is over there is little to be done in most cases except to provide for warmth and rest. The former is supplied by hot bottles, one at the feet and others on each side of the patient. In the case of a baby it is well to place its cradle in front of the fire during the period of shock. The rest which has been mentioned must be as perfect as can be, being undisturbed by frequent examinations of the pulse and other unnecessary interferences. But this does not mean that the patient is to be neglected. His wants must be anticipated,

Hourly Temperature Chart, Showing Marked Fall of Temperature Following an Operation of Moderate Severity.

and his comfort attended to with as little disturbance as possible; indeed, rest may be impossible for him unless this is done.

### Saline Injections.

There is one remedy which is of great service in combating the depressing effects of a severe operation, and this is the injection of normal saline solution at a temperature of 105° F., either by the bowel or under the skin. It is a curious fact that in shock the blood becomes more viscid, and it is possible that saline injections owe their beneficial effect, in part at least, to a reduction of this viscidity. But whatever the explanation may be, there is no doubt about the efficacy of the injections for relieving shock. The methods of administering saline injections will receive attention in a future article.

In shock the circulation is feeble, and consequently the patient's blood tends to accumulate in the veins, and especially in the large veins of the abdomen. To counteract this tendency it is sometimes recommended that the foot of the bed should be raised, and the abdomen tightly bandaged. The foot of the bed may be raised either by placing suitable blocks under the legs, or by resting the foot of the bed on a chair, or on two chairs if the bed be a wide one.

Drugs are of secondary importance in the treatment of shock, with the exception, perhaps, of morphia and opium. But some surgeons have faith in strychnine, alcohol, especially in the form of brandy or whisky, and adrenalin.

Factors complicating Shock.

There are certain conditions which, although physiologically distinct from shock, are frequently found in association with this condition, and it may be worth while to mention them so as to make a clear concept of shock possible.

Acute anæmia and syncope are examples. The former is not infrequently an associate of shock. Rupture of an ectopic gestation sac is an example. The hæmorrhage complicates the shock, but the two processes are distinct. The anæmia caused by the loss of blood leads to a diminished circulation of blood in the brain, and consequently the nerve centres are more easily exhausted than they would be otherwise.

Summary.

Secondary shock is due to exhaustion of the vital nerve centres by repeated stimulation of sensory nerves. Its treatment is chiefly preventive, and consists (1) of improving, when possible, the general condition of the patient, and consequently increasing the resistance of his nerve centres; (2) of lessening the sensory stimulation by therapeutics, and especially by early operation (which in septic cases also puts a stop to septic poisoning); (3) by keeping the patient warm. When secondary shock is already present it is to be combated by (1) rest, (2) warmth, (3) saline injections, and (4) drugs, especially opium.

### The Nurses' Clinic.

### THE DISTRICT NURSE AND THE PREVENTION OF PUERPERAL FEVER. BY MISS M. LOANE.

In district work I cannot honestly say that I have ever known cases where severe puerperal fever has been cured, but much can be done by timely and detailed advice to prevent its occurrence. When midwifery is undertaken by the district nurse there should be such a regular system of booking the cases beforehand that any unexpected calls upon her attention are the rare exception. Three months before the probable date the expectant mother should call on the nurse, give her full information as to her circumstances, previous confinements, etc., and receive full instructions from her as to the preparations to be made. Three weeks before the event the nurse should visit the patient, ascertain how much has been done, or is about to be done, and, if necessary, repeat her instructions. The one word "cleanliness" sums up the whole, but unless the nurse enters into particulars cleanliness, even in decent homes, may be overlooked in some important respect.

*Attendance.*—The first point to be insisted on is that the visiting nurse will not undertake the case unless a respectable relative or neighbour is engaged for at least ten days "to keep the house going," cook for husband and children, do the washing, and give necessary attendance to mother and infant between the nurse's visits. This person should, if possible, be not less than thirty, nor more than sixty years of age, healthy, very clean in her person, willing to wear a cotton blouse and a skirt of some kind that will not hold much dust, and, when attending on the patient, to wear a large clean apron, tuck her sleeves up well, wash her hands and arms thoroughly, and keep her nails short and free from black rims.

*House.*—The house should be thoroughly cleaned throughout before the confinement takes place. All respectable women are in the habit of doing this, not from fear of infection, but in order that the household may suffer as little inconvenience as possible while they are laid by. In most cases, therefore, it will only be necessary for the nurse to draw attention to such points as the cistern and the drains. The dustbin should be cleaned out thoroughly and disinfected, the sink well scrubbed and kept clean, the w.c. scrubbed, and the walls limewashed.

*Patient's Room.*—Special attention must be given to the patient's room, which must be prepared as carefully as if for an operation; the strips of carpet should be taken up, beaten and washed, the floor and all the woodwork washed with carbolic. (One tablespoonful of crude carbolic to be stirred in with a stick to two gallons of warm water. Carbolic can be obtained gratis from Town Hall or Vestry). The walls must be swept, the pictures and ornaments dusted and washed, the furniture cleaned. The framework of the bed should be thoroughly washed and completely freed from dust, and the mattress covered with well-washed and boiled unbleached calico. During labour the mattress should be protected with three sheets of brown paper, a piece of sacking, and a drawsheet well pinned with safety pins to the mattress. There must be at least three pairs of clean sheets, several old ones for draw sheets, three or four pillow cases, and the blankets should be as new as possible, in order that they may be warm in proportion to their weight. All needless curtains and valances should be removed, and the remainder should be of washing materials. The blind must be clean and in good order. Careful consideration must be given to ventilation, remembering the dread that the poor have of fresh air at these times, and the terrible results of catching cold. A small fire, a Hinckes-Bird ventilator, and a clothes-horse screen will generally meet the requirements. Whether a fire is needed or not the chimney should be swept, as the lining of soot in the chimney is not only an excuse for closing the register, but interferes with the ventilation when it is left open.

*Patient's Clothing.*—The patient must undress (in backward districts this is postponed until the child is actually born) and wear a clean chemise and nightdress pinned up under the arms until after the birth of the child, and a clean flannel petticoat, which will be removed when the other garments can be drawn into place without risk of staining. Soiled garments must be taken off at once, but moving the patient about much is so undesirable that every care must be taken to avoid the necessity. At least a dozen large soft diapers will be required. They must be removed, like other surgical dressings, directly the discharge comes through, but a piece of sterilised absorbent wool covered with a piece of sterilised butter muslin or cyanide gauze can be placed next the vulva. The diapers should be fastened with safety-pins to the binder, as they can then be changed without much disturbance. At least three binders, 18 inches by $1\frac{1}{4}$ yards, will be needed. They should be made of huckaback, washed and boiled. Two clean, soft shawls should be in readiness; if not, a poisonously dirty one will very probably be wrapped round the patient the first time she complains of feeling cold.

*Personal Cleanliness.*—The patient must be warned to

**THE NURSES' CLINIC**—*Continued.*

keep herself as clean as possible all through the period; one sudden bath at the end might well do more harm than good. The danger of self-infection, if cleanliness is neglected, must be carefully pointed out. The patient should be warned to have her hair very clean and well brushed and plaited, no hair-pins being allowed while in bed.

*Table.*—A table must be placed by the bedside with sterilised threads for the cord, sterilised squares of old cambric or cyanide gauze, a pair of blunt-pointed scissors, everything necessary for giving a douche, and a basin of lotion (mercury 1-2000) for the nurse to dip her hands in (hands having first been thoroughly cleansed).

*Placenta.*—The placenta must be placed in cold water and carefully examined to see if it has come completely away. Shreds of the placenta left in the uterus may easily cause puerperal fever. When the uterus has become sufficiently hard, put on the binder, take temperature and pulse, move the patient to clean half of the bed, take away all soiled linen and place in cold water and disinfectant. The patient should then be given milky tea with a beaten-up new-laid egg in it and allowed to sleep. The uterus should be kneaded by the nurse twice a day for nine or ten days when she changes the patient's binder.

*Temperature.*—The temperature and pulse must be taken twice daily as long as the nurse is in attendance. Any temperature above 100° F. is suspicious, and rapid pulse is a still more serious sign. It must be remembered that puerperal fever often begins on the second day, and the earlier it can be detected the more hope there is of cure. The vulva must be washed twice a day with two pieces of clean boiled rag dipped in creoline and warm water (one teaspoonful to one pint), and wiped with another piece of dry rag, all rags being afterwards burnt and the lotion basin scalded out and dried.

### The Nurses of Kingston Union Infirmary.

INTERVIEW WITH THE MATRON. BY OUR COMMISSIONER.

Three years ago last March the handsome Union Infirmary at Kingston-on-Thames was opened by special order of the Local Government Board; but the training school for nurses was commenced some little time previously by Miss J. A. Smith, the first matron. The new infirmary is more than a local institution. It serves seventeen parishes, including Wimbledon; its 395 beds, most of which are always full, are therefore often occupied by patients coming from a very considerable distance. On the occasion of my visit, a few days ago, before I began to talk to the matron about the progress of the school, she showed me round the whole of the wards and quarters of the nurses, who are located in three separate buildings. There is an obvious inconvenience in regard to the latter arrangement, but fortunately there is ample space for the enlargement of the new home in which the bulk of the staff are already housed; and in due time, when the guardians feel that they are justified in incurring the necessary expenditure, there is no doubt that the nurses will be accommodated under one roof. With this exception, the provision made both for the patients and the officials appears to be all that could be desired; from the spacious, lofty, well-lighted, well-ventilated wards, to the extremely comfortable sitting-rooms of the sisters and probationers. As we made our way round the wards the matron said, "There are seven floors and each sister has charge of one floor. The three male wards are known as A, B, C, and the four for the females as D, E, F, G."

"How many probationers are on duty in each ward during the day?"

"Three, and one during the night. In the event of any special treatment being required a special additional probationer is provided. With regard to night duty, the night superintendent and the assistant, who is also a fully trained nurse, are fixtures, but the probationers are on duty at night for three months only. I should add that on floors D and G, which include the wards for maternity cases and children, the former being at the top of the building, there are two probationers on duty during the night. I never put any probationers on duty at night until they have been here four, five, or six months."

### Probationers on Trial.

"Of course they come to you on trial?"

"Yes, for three months, and during that time they work in the wards, but are not included on the staff. I attach great importance to the trial; probationers find out whether they like the work and I ascertain if they are suitable for it. During the three months each sister furnishes me with a written report as to the manner in which the candidate performs her duties—these reports influence me in my decision, but, of course, I judge a good deal by my own observation."

"How many sisters are there?"

"Eight day sisters, one home sister, and two night sisters. There is also an assistant matron. The home sister is in charge of the nurses' home, by which I mean the new building, and all apartments occupied by the nursing staff and maidservants. She also assists me in presiding at the nurses' meals, and looks after the servants."

"What is the average number of probationers?"

"Thirty-six; though my Committee are very good and do not limit me if I require more. The age considered desirable is from twenty-one to thirty, but I try to have them from twenty-three, if possible. The period of training is three years and the salary is £10 for the first year, £15 for the second, and £20 for the third, with indoor uniform. During the winter, lectures are delivered once a week by the matron and assistant matron on nursing and bandaging; by the medical officer on midwifery, and by the assistant on anatomy and physiology. Sometimes the medical officer gives lectures on diseases. I am very particular that the probationers should take care of their own health; they are not allowed to be absent from meals or to go on duty before they have had breakfast, and I exclude from off-duty time any portion of the meal hours."

### The Hours of Duty.

"When do the day nurses enter the wards?"

"Probationers 7.30 A.M., sisters 8 A.M., winter and summer alike. They leave at 8 P.M., have supper at 8.15, go to bed at ten. Ordinarily, no nurse is allowed out after 8 P.M., but the probationer in her third year has one evening weekly from 4.30 to 10, and all the probationers have a whole day off once a month. Sisters have an evening off and the next day as well once a month; this gives them a night at home. The daily recreation is from 2.30 to 4.30, or 4.30 to 7. On Sunday half the staff are off duty from 10 A.M. to 1 P.M., and the other half from 2 to 10 P.M., alternately. As to holidays, the probationers in their first year have two weeks and the others, with the sisters, three weeks."

"How about night nurses?"

"In summer they are called at 4 P.M., breakfast at 4.30, are out from 5 to 7.30, when they have dinner. They are due in the wards at 8.15 P.M., leave at 8 A.M., have supper at 8.15 and are expected to sleep from 9 A.M. to 4 P.M. In the winter the hours vary a little. Their recreation time is from 9 to 11.30 P.M., and on Sunday they are off alternately either from 9 to 12.30 or from 5 to 9. Nurses who desire to attend early service on Sunday are permitted to do so, at any of the churches outside the Infirmary; we have no chapel and in any case I think that it would be better for them to go elsewhere."

### The Nurses' Quarters.

"Although your nurses' quarters are not all under one roof there seems to be as far as possible every provision for their comfort?"

"It is usually thought that they are fortunate in having pleasant and home-like sitting-rooms. As you have seen, it is better to have them there than in the main building. When the nurses get their day off they have their breakfast in their own bedroom, and if they are out on late leave they report themselves to the matron or assistant matron. Sisters dine at 7.15 instead of having supper. It is one of the advantages of having all meals included in the duty time that there is no rushing away from the table to catch trains."

### The Pension Fund and Christmas Presents.

"Do many of your staff belong to the Superannuation Fund?"

"Only two or three. There is no compulsion in the matter. It is easier to keep up the payments to the National Pension Fund than to the Superannuation Fund. Several of the staff, as well as myself, belong to the Pension Fund, and more have joined since the meeting here."

"It would be interesting to know your views about Christmas presents."

Some of the Nurses at Kingston Infirmary.

the bedrooms of the sisters are rather larger than those of the probationers, but the furniture is much the same."

"I notice that all have fire-places."

"Yes, I consider that fire-places are essential for ventilation, and for use in cases of indisposition. But permission has to be obtained to light a fire. The home sister looks after all sick nurses, though I am glad to say that ours are seldom unwell. I think, however, that staying in bed for a little time often prevents illness. A radiator outside keeps the passages warm, and the electric light is provided in each room. As to the latter, great care is taken to avoid waste."

"The nurses," continued Miss Smith, "are under no temptation to have dangerous spirit lamps in their rooms because there is a gas stove on each landing in the home at which they can make tea if they like. Tea, by the way, is always provided for those who wish to have it at supper."

"Where do the nurses have their meals?"

"They are all served in the dining-room at the home, it

"Our great idea at Christmas is to make the place as home-like as possible and to keep up the real spirit of the season. One shilling is allowed by the guardians for toys for each child (the average number of children being 35), and £2 10s. for flowers and vases. We sometimes receive gifts of toys, plants, and flowers from the public. The sisters and nurses find the rest of the money for decorations. They all like to give presents, but it is the spirit of giving rather than the actual gift that we set store by."

"Do you get plenty of applications for admission to the training school?"

"There is scarcely a day passes without my receiving one; but, of course, the applications are not all suitable."

"You appear with your up-to-date operating theatre, your well-fitted-up maternity room, *x*-ray room and radium, and your balconies for the phthisical patients, to enjoy most of the advantages of a general hospital?"

"With none of the drawbacks of a medical school. Seventeen of our probationers passed the L.O.S. examination and now hold the certificate of the Central Midwives Board; and several nurses who were trained here are now our sisters. Others are sisters at Bradford Infirmary, Bolton Infirmary, the National Hospital for the Paralysed, and staff nurses at Surgical Homes in London, and on the staff of Queen Alexandra's Imperial Military Nursing Service. The Committee give me a free hand in respect to diet, and as long as I do not exceed the amount allowed, I can order what I like. We have an adequate staff of proper domestics, not wardmaids, but paid scrubbers; also of cooks, housemaids, laundry-maids, and porters."

"I think that you were trained at Birmingham Infirmary under Miss Gibson?"

"Yes, and subsequently I was ward sister and obstetric sister, being there altogether for six years; I then went to Bradford Union Hospital as superintendent of nurses for three years, and there I organised a training school. From Bradford I came here to Kingston. As you know, we started a Nurses' League here last March. It is perfectly independent, and the members are not committed to the support of registration. Altogether, I think it may be said of Kingston Infirmary that things are comfortable but not extravagant, and that there are indications of progress all round.

### Incidents in a Nurse's Life.

Contributions to this column are invited.

### OUR CHRISTMAS WEEK.

As December came to an end the patients in the Cottage Hospital one after the other asked to go home, as they were better, and so it came that I, the Matron, and the probationer were left with only two patients, two girls, who were obliged to stay. So I planned to take a half-day off, which I had not had for weeks, and settled to go a long walk to a village nearly four miles off. Just as I was ready the Rector of that very village called in his pony trap and offered to drive me out, so I accepted, and started off. The Rector's wife gave me lunch and asked if I would mind going to see an old woman in the village who was very ill. So I went, inwardly wondering whether my half-day off would be as refreshing as I had hoped if "cases" were thus introduced into it. I found the poor old woman living in a small cottage alone. It was very clean and tidy, but she was very ill, and I discovered that she had a strangulated hernia, and had had constant fæcal vomiting for hours. I gently tried to get the hernia back, but it would not move, so I said she should see the doctor, and meanwhile I suggested the constant application of cold-water cloths to the hernia. As one of the ladies from the Rectory volunteered to remain, I showed her how to apply the cold flannel, and made a pad of flannel round the hernia, to prevent the water soaking through. I then wrote a note to the doctor, and, leaving it on my way, hurried back to the hospital. When the doctor went out to the village he found the hernia partly reduced, the fæcal vomiting stopped, and the patient fairly easy; but he arranged for her to come into the hospital next day. Meanwhile, when I had arrived at the hospital gates there stood a cab, and a man was being helped into the hospital. Here was a new patient! So we got him to bed; he had influenza and an inflamed tendon of the leg. We were rather tired when we went to bed that night, but at six in the morning we were knocked up. A man had broken his leg, and had lain on the road all night in a bitter frost. So, of course, we took him in, and later in the day came the hernia patient in the carrier's van.

This was on Christmas Eve, and in the afternoon the two doctors came and operated on the hernia, and as they were both needed, I had to go on with the anæsthetic. The hernia was transfixed by a small piece of bone; it was over an inch long, and seemed to be part of a rabbit bone, evidently swallowed in broth. It had pierced the gut, and gangrene had begun, so there was no hope for her recovery. I remained with her all that night and all the next day (which was Christmas Day), and still on and on. Nothing eased the vomiting; yet she had no pain; she vomited every three or four minutes; at last I felt tired out, and was obliged to go and get some sleep. It seemed so selfish to go and leave her; but I had been up now 33 hours on end. However, I made my probationer promise to fetch me if there was any change. At 5 o'clock the doctor came again; and said I was to be called. The patient knew me, and put her hand in mine. She had often said she was glad to be with us in our little hospital, and not in the Union, to die—and so she died. She was only a poor old woman, yet the doctor sent a wreath from his garden to put upon her coffin.

It was over so soon; no relatives came until after, and the first one was an old patient—my embolism patient, an account of whose long illness has been given before. He was the poor woman's nephew, and as he silently wiped away a tear he said, "Poor dear, she could not have died in a better place."

So our Christmas was over.

### Central Midwives Board.

### THE REPORT OF THE STANDING COMMITTEE.

The meeting of the Central Midwives Board, which was held on Thursday afternoon last week, occupied scarcely more than half-an-hour, the chief business being to receive the report of the Standing Committee. There were present: Dr. Champneys (chairman), Dr. Dakin, Miss R. Paget, Miss Wilson, and Mr. Parker Young.

Mr. Parker Young moved, Dr. Dakin seconded, and it was carried that those parts of the letter from Dr. Fothergill on behalf of the examiners of the Manchester Centre which concerned the examinations directly, be sent to all the examiners for their consideration and report, and that the whole letter be dealt with after their opinion had been received.

The report of the Standing Committee was received and accepted. The Committee reported that with regard to a letter from Dr. A. Mearns Fraser, Medical Officer of Health, Portsmouth, in reference to the finding of a *prima facie* case of negligence and misconduct against Georgiana March, certified midwife, the Secretary had written for further particulars but had had no reply; that Caroline Harvey, certified midwife, against whom the Clerk of the Derbyshire County Council reported the finding of a *prima facie* case, was recommended for censure; and that further information had been requested concerning Mary Ann Betton, against whom a *prima facie* case had been found by the same authority, but that no answer had been received.

Jane Tween, of whom Dr. Thresh reported negligence and misconduct, was cited.

Mary Ann George, who was reported by Dr. Read, Medical Officer of Health at Worcester, to have been in January 1905 convicted of larceny and on October 27, 1905, convicted and fined twenty shillings for having failed to notify the Local Supervising Authority of her intention to practise as a midwife, was cited. Mr. Parker Young desired to call special attention to the action of the Rev. F. H. Richings, Rector of St. Clement's, Worcester, in regard to this case. Mr. Richings signed a certificate of good moral character for the woman, within two months after her conviction for larceny and with apparent knowledge of her conviction. His reply to the Board, on their communicating later with him, was to the effect that he believed her to be at the time of his signing the certificate of good character, and he was under the impression that he had used the word "now" or otherwise qualified his statement. The Secretary produced his certificate and it was shown that no such qualifying phrase had been used.

With regard to a letter from Dr. Newsholme, Medical Officer of Health at Brighton, reporting a fatal case of puerperal septicæmia occurring in the practice of a certain midwife, and asking the opinion of the Board thereon, it was decided that there was no case, as several doctors were called in and disagreed in their diagnosis of the case.

Hannah Howe and Esther Smith, certified midwives, were cited on the report of the Clerk of the County Council of Durham of the finding by the Local Supervising Authority of *prima facie* cases of negligence and misconduct.

It was decided to recognise as training schools the Kensington and the Fulham Union Infirmaries, as the defects had been remedied for which the recognition had previously been delayed.

A letter was received from the Clerk of the Staffordshire County Council reporting the suspension from practice of Eliza Pugh, certified midwife, No. 1,756, on account of her connection with a case of puerperal fever, and recommending the removal of her name from the roll.

The midwife was cited.

January 30 was fixed for the hearing of the charges against Elizabeth Jacklin, certified midwife.

The Secretary was instructed to reply to a letter from the Clerk of the Lambeth Guardians asking the reason of the Board's refusal to approve their Workhouse Infirmary as a training school, and to state that the chief reason was that the Infirmary was considered structurally unfit.

The recommendations of the Committee with regard to applications for recognition as institutions under Section C of the rules and applications for approval as teachers and midwives were adopted.

It was decided to send out to the Board's inspectors the form of directions, suggested by Miss Wilson, with the amendments proposed by various members of the Board.

Owing to the absence of Mr. Ward Cousins his proposal with regard to a form of suggestions for the guidance of the medical advisers of the Local Supervising Authorities respecting the duties of inspectors, was postponed.

The Secretary reported that it was necessary to revise the Rules before Easter and it was decided to hold a meeting as early as possible for that purpose.

Miss Wilson brought up the case of the Cheltenham Nursing Home again before the Board. Considerable hardship, she stated, had been caused to four nurses who, owing to the change of date in the examination, would be unable to sit for the forthcoming examination, unless the Board relaxed their rules and allowed their forms to be filled in less than three weeks before the date of examination. The Chairman pointed out that not only would they commit an illegality by relaxing the rules, but that they would also be rescinding a previous resolution, which could not be done without fourteen days' notice. Miss Wilson, therefore, gave notice of a motion on this point for the next meeting, as she held that the circumstances were peculiar, since it was due to the Board's own vacillation as to dates that the difficulty had arisen.

### The Hospital for Sick Children.

### NURSING OF INFECTIOUS DISEASES.

A new department for the reception of cases requiring isolation has just been completed at the Hospital for Sick Children, in Great Ormond Street, and thanks to the courtesy of Miss Payne, the matron, our representative has had an early opportunity of inspecting it. The department occupies an entire floor, comprising four small, one large ward, two bedrooms for the use of nurses on duty, lavatories at each end, two bathrooms, and a spacious kitchen. The whole is so fitted and arranged with every need and convenience to hand that, while insuring complete isolation, the work can yet be carried out on the same basis as the rest of the hospital. Each small ward contains one bed; three are intended to receive patients and one nurse during a "suspicious" period, and the patients will remain in these wards until the rash or other symptoms are sufficiently developed to be diagnosed. Scarlet fever is not nursed in the hospital, and cases of diphtheria and measles are removed to the special diphtheria and measles block. One of the three small wards, built at the further end of the corridor away from the others, is for the reception and nursing of cases of erysipelas.

The larger ward, which contains eight beds, is a "change ward" receiving patients while the general ward is being disinfected after an epidemic has occurred. The general colouring of wards and passages is green, the floors are covered with brown linoleum, and the tables with bright red American cloth which makes a pretty cheerful note. The fireplaces and chimney-piece are built of green tiles, which, with the firelight playing on them, give a charming and homelike feature. The various doors are made of solid teak effective and fire-proof. Electric light prevails throughout. The department is situated on the fifth and last story of the hospital; fortunately a new lift has recently been acquired, so that time and trouble are saved. The lift can be worked by the nurses and has an admirable safeguard against accidents—being so arranged that the entrance door can only be opened when the lift has reached the floor on which it is required.

Opening out of one of the isolation wards there is a delightful large balcony, or rather open-air play-room, built on the roof of a ward below, which is to be fitted up later with chairs and lounges. It is safely fenced in with wire, and being so high up the air is most exhilarating, while on a clear day a very extensive view is to be had, to say nothing of sky and sunshine, which will doubtless prove a pleasure as well as a benefit to patients and nurses alike.

The old isolation ward will in January be turned into an additional surgical ward.

### Presentations.

Nottingham Nursing Home.—On the occasion of the resignation of Nurse Hutchinson, who is giving up nursing owing to her marriage, the matron of the Nursing Home, 19 Regent Street, Nottingham, gave a farewell tea. Miss Hutchinson was presented by the nurses with a large picture by Marcus Stone entitled "The First Love-letter," and she was also the recipient of a silver tea-caddy from the matron.

### Wants and Workers.

Will anyone kindly help a district nurse by sending some left-off underclothing or useful garments to Nurse Asplen, 14 Myddleton Road, Uxbridge?

### Visiting-Nurses in the United States.

By Evelyn W. Jefferson, Superintendent, Instructive Visiting Nurses, Washington, D.C.

Reading a copy of the Nursing Mirror lately it occurred to me that perhaps English nurses might be interested in the methods of some visiting nurses in the United States and the patients they have to deal with.

### The Washington Branch.

As yet there are few Societies of Visiting Nurses who have a central home—indeed there are not many Institution Visiting Nurses' Societies at all; here and there, in the West, may be found a solitary nurse, "an experiment," one is told. In Washington, D.C., the national capital, we have been established since 1900. Through the untiring efforts of Miss Emily Tuckerman, a lady well known for her interest in wise philanthropic movements, a nurse was placed in the slummiest section of the city. Her work was so excellent, showing such great need, and making such demands on her, that in May—just three months later—another nurse was started, on Capitol Hill. In June, so rapid was the growth of interest, that a third nurse was put in the north-east, and in July a nurse was established in the old part of this city—Georgetown. Since then we have added three more and a Superintendent. We live in a home, have two coloured servants, and, for a houseful of women of different nationalities, live very happily. Our work begins at 8.30 a.m. and ends when we get through—any time, in winter, from 4.30 to 7 p.m. Each nurse has a "loan cupboard" and invalid cots and chairs at her office, which is situated in the district to which she belongs. Her calls come from the physicians to the poor, clergy, policemen, in short, anybody who thinks they need her services. Most of our cases are charity, but those who can are encouraged to pay a small fee, if only five cents—twopence half-penny—or less each visit. Often some of the coloured women will do extra washing returned in bad condition from other patients. We work irrespective of colour or creed. We often find ourselves in most unsavoury houses—in two ways unsavoury, in odour and in reputation. But we have never yet required police attendance.

### The Negro Patients.

I often wonder what English nurses would say to some of the negro patients. The average negro is the most irresponsible, dirty, lazy, shiftless, and amazingly immoral personage; nobody who has not lived in the more southern States can realise what they are. Many are the old darkies who tell you, "De cullud people, miss, noht what they war, no, indeed honey, deys that impident," and so forth. Others are proud to show their manumission paper. They make good patients if you keep the upper hand. There are splendid examples of their race among them, but the average negro is better if treated more or less like a child. A smile or joke, or a good sound "dressing down" at times, will do more good than polite treatment as equals. It is a fact that Northern people are very often distrusted and disliked because they treat the negro as an equal; indeed, I have often seen them really despised for according such politeness in even giving prefix of Mr. or Mrs. English people are apt to get on with them because they are used to a dependent and menial class. It must be remembered that these remarks do not apply to the whole race, only to the average individual—so many of whom are met in Washington, the coloured man's Mecca. I could write pages of stories about our coloured patients; we have almost always found them grateful and obedient.

### Practical Results.

We instruct as much as possible, and often find, on a call, perhaps coming months after the first time, hot water, clean floors, aired bed linen, and even occasionally raised windows, showing our former work has not been in vain. While not getting the same class of foreigners as seaport cities, we have many Jews, Syrians, and Italians to deal with, and also a peculiar and vicious class of low whites—real "white trash." They are the lowest order of beings, and may be of any nationality. There are none more absolutely hopeless to deal with.

### The Hours of Duty.

Washington has a particularly trying climate. As the houses are not built for heavy winters, these last three severe ones have been productive of much suffering and distress. The mortality from tuberculosis is great, while in summer many are the deaths from cholera infantum. It is hard to teach effectively the need for clean babies and proper nourishment rightly prepared and administered. We are entirely supported by voluntary contributions. The nurses receive 25 dollars per month (£5), uniform, board, lodging, and allowances for laundry and car fare. They get a half-day per week if allowable, and take none but emergency cases beyond their regular necessary visits on Sunday, but especially in winter it is rare to get two consecutive Sundays without making at least two important calls. We do not work at night, and our Board wisely decided that we were to take no calls at night, for working women cannot burn the candle at both ends. Sometimes we may have a night nurse of our own. Meanwhile for bad cases we employ caretakers, or accept the voluntary assistance of a Sibley deaconess or free private nurse willing to volunteer.

### School Work.

Last spring we began our school work. That means, the children excluded from each school by the medical inspector are followed up by the nurse in whose district they live, and either sent to dispensaries or to the family physician for instructions and treatment. The work is as yet not well systematised, but by spring we hope to have things reduced to a more practical working plane than is now in operation. Perhaps it may add to the interest of English nurses if I add that I, the writer, am an Englishwoman.

### Open-Air Treatment of Phthisis in India at a Hill Station.

Where I am nurse is at a favourite hill station, standing 7,000 feet high, and we have patients sent to us even as far away as from Rangoon. We admit not only phthisical cases into our hospital—which is quite a small one of twelve beds—but all kinds of cases, surgical and medical, many being enteric. The latter, I am glad to say, we have been very successful with, last season having had twelve or thirteen cases, mostly acute, and they all recovered. The doctor in charge of the hospital is very keen about the treatment of phthisis; he has open verandahs just enclosed with wire netting, where the patients sleep during the rains. Only those who have experienced what the rains of India are can fully realise the amount which can fall in a short space of time. As a rule the rainy season lasts for nearly three months with almost continuous heavy downpour, so that it becomes rather depressing. You go to bed with the sound of rain beating on the corrugated roof, and you wake with the same sound in your ears; but there are compensations in seeing everything fresh and green around you, and the ferns on the sides of the mountains—and even growing on the trees—are simply lovely. Before the rains set in the phthisical cases all sleep right outside in the open, and it is a funny sight if a bad storm gets up—and we get them very severely—to see the night nurse bundling in mattresses, bedding, etc., and the patients following in her wake. At first they are rather inclined to shiver and complain that it is too cold, but before long they are quite disappointed when told the weather is too bad for them to sleep outside. One of these patients was given only six months to live by two or three doctors. She came to us in April; it is now June, and she is so much better that she does everything for herself and goes for a walk of half to three quarters of an hour every day, and her temperature has remained normal for the last month.

Another, quite a young girl, was also given only a short time to live. Her temperature on admission was from 102° to 104°, pulse 140, respiration 36. After a few weeks' treatment it settled down, and now it is never above 100°, pulse about 112 to 120, and respiration 20 to 24. She came to us in the middle of April, and was kept absolutely quiet in bed for 2½ months. Now she gets up and walks about a little in the open air, and has put on about a stone in weight.

The patients are on full diet, with two pints of milk in twenty-four hours. The meals are: early tea and toast 6 A.M., breakfast 9 A.M., porridge or some similar milk dish, meat, eggs ("dhal") and rice, or kedgery, tea made chiefly with milk, and bread and butter *ad lib.*; 11.30 A.M. glass of milk.

Tiffin (lunch) 2 P.M., hot meat, potatoes and whatever vegetables are in season, milk or suet puddings, stewed fruits, custard, etc., and glass of milk. Tea, 5 P.M., with bread and butter. Dinner, 7.30 P.M., soup, meat, or chicken and pudding, with a glass of milk. Fish can hardly be obtained here, or else it is too dear to buy, and the Indian chickens are so small that they are not much bigger than a grouse. They range in price from 4 annas (4d. in English money) to 12 annas (1s.). The patients are compelled to eat as much as they can, and even if they feel sick they are not let off anything. Certainly the "stuffing" process seems half the battle, and it is quite wonderful how quickly they are able to eat nearly double the quantity they can manage when they were admitted. Of course, the hill air has something to do with the improvement in their appetites, but also people get into the habit of eating as much as they can. Even when the weather is very cold the patients only wear cotton night-gowns and no under-vests; but they are allowed as many blankets as they like during the night. I wish that all who had a tendency to consumption could undergo this open-air cure. I have seen such good results from the treatment.

### Resignations.

DULWICH HOUSE CONVALESCENT HOME, CARDIFF.—Miss B. A. Hope, who has been our matron since the opening of the Dulwich House Convalescent Home, Cardiff, has resigned her appointment. She went to Cardiff from the Manchester Children's Hospital, where she had held the posts of night superintendent and ward sister. She received her training at the Manchester Children's Hospital and at Guy's Hospital, London.

### "The Hospital" Convalescent Fund.

THE Hon. Secretary begs to acknowledge with grateful thanks the receipt of 10s. from an anonymous contributor at Edinburgh; 10s. from E. A. H.; and 2s. 6d. from the Travel Correspondent.

### Everybody's Opinion.

[Correspondence on all subjects is invited, but we cannot in any way be responsible for the opinions expressed by our correspondents. No communication can be entertained if the name and address of the correspondent are not given as a guarantee of good faith, but not necessarily for publication. All correspondents should write on one side of the paper only.]

### BREAD CAST UPON THE WATERS.

"AN EDINBURGH NURSE" writes: "Will you kindly allow me to acknowledge in your columns the sum of 2s. 6d. which was sent me for Mary, the little girl in the story headed "Bread cast upon the waters," which appeared in your issue of December 2. No address was given me."

### MENTAL PATIENTS. NIGHT AND NUDITY.—A DURHAM SCANDAL.

DR. P. D. HUNTER writes from Durham County Asylum, Winterton, Ferryhill: "I am exceedingly pleased to see that you have published our matron's letter protesting against her dismissal. As the dismissed medical officer I have hitherto refrained, on the advice of friends, and entirely against my own inclinations, from appealing to the public Press; but I agreed with them that the matron as an injured woman was more likely to win sympathy than myself, and I am only waiting to criticise any statements or misstatements that may be made in attempts to explain or refute any words of the matron's letter. The local Press is apparently chary of voicing the grievances of individuals against a public body, but the injustice to the matron at least has been so gross that I feel that in publishing her statement you are but lending her that freedom of the Press which is our country's boast. I may have to say more on my own case as occasion offers; at present I can only repeat my grateful thanks that you have taken the course you have."

### THE LONG VEIL.

"OLIVE" writes: With regard to the vexed question about who should, and who should not, wear the long veil, which is almost always recognised as part of the uniform of a hospital nurse, the best way to settle the matter once and for all would be for the Matron of every hospital in the Empire to bring up the matter before the management, and ascertain their views on the subject. If they are in favour of their nurses wearing an outdoor uniform, then let them have a badge with the crest and motto of town or city in which the hospital is situated, prepared in such fashion that all who have attended the first year lectures given by the doctors, and passed an examination if required, can wear the badge pinned on to the left side of their cloak. No nurse worth her salt would object to let the whole world see that she identifies herself with her training school, and that she is proud of being one of the staff. It is absurd and altogether out of place that domestic servants who become nurses to the children of private people should wear a veil at all, and I am more than astonished that their employers allow them to do so, apart from the fact that it is a dangerous article of head gear on a windy day, when it requires all a nursemaid's attention to attend to her young charge. I hope that some persons in authority will take this matter up and decide for or against.

### AN EXPERIENCE IN EDINBURGH.

"DISTRICT NURSE" writes: Every day we keep getting new cases, and we never feel happy till we see what our new patient is like. Not long ago I was asked to visit a "poor old woman" who was suffering from rheumatism and a burn in her foot. I had a busy morning, and did not get my new case in till late in the afternoon. Arriving at the door I knocked, and was asked to come in. I opened the door and found my patient sitting in a corner beside the fire in great pain. "Oh, nurse," she said, "I was beginning to think you were not coming; how I have been longing for you"! I asked her why she was not in bed, and on looking round the little room I noticed that her bed was made up with boxes and boards, and very little blankets or coverings. She said, "Well, nurse, it is warmer at the fire." Then I asked her if it was not lonely for her to be living alone. "Oh! no," she rejoined, "You see I have the little bird, Jenny, and she is a great companion to me. She sings and speaks, and she knows I have this burn in my foot and has been sympathising with me to-day. When I am reading the lesson Jenny joins me. No, nurse, I never weary or feel lonely." I put a dressing on her foot and left her, saying that I should call some time the next evening. I was glad to go again the next evening, and found my patient with only a faint light coming from the little fire. "Sitting in the dark," I said. "Yes, but I see fine for all I am doing; but there is a candle in that corner for you, please get it, nurse." Then she began to tell me that she had had a better night, and her foot was not so painful, but Jenny the bird had a cold, and she was covering the cage with old clothes to keep Jenny warm in the night. She said, "Nurse, I should not like anything to happen to my little bird." "Poor old body," I thought, "you really think more about the bird than yourself." Then I asked her if she were happy. "Yes," she said, "you know I just ask for a blessing, and I always get it." I left her thinking how lonely her life seemed to me, and yet she was perfectly happy and contented with very little. The burn is better now, but she is still suffering from rheumatism.

### Appointments.

Basford Isolation Hospital, Nottingham.—Mrs. Mary E. Byrne has been appointed sister. She was trained at Shoreditch Infirmary and has since acted as staff nurse at that institution.

Bradford Infirmary.—Miss Mary Anne Lloyd has been appointed theatre sister. She was trained at the Tottenham Hospital, London, and is a member of the Meath Workhouse Nursing Association.

Chesterfield Union Infirmary.—Miss S. Heap has been appointed staff nurse. She was trained at Bromley Union Infirmary and has done temporary work at Birkenhead Union Infirmary.

Crossley Hospital, Mirfield, Yorks.—Miss Lucy Acomb has been appointed matron. She was trained at the Leeds Infirmary, and was afterwards nurse at the Cumberland General Infirmary, Carlisle, where she acted as night sister *pro tem.* She has since been charge nurse at the Brook Hospital, London, and sister and deputy matron at the Sanatorium, Huddersfield. She has also taken matron's holiday duty at Whinlatter Isolation Hospital, Cumberland.

Dulwich House Convalescent Home, Cardiff.—Miss Mabel W. Fox has been appointed matron. She was trained at Manchester Northern Hospital, where she was afterwards sister. She has since been sister at the Sea Bathing Infirmary, Scarborough, sister at Nottingham Children's Hospital, and sister at the Jenny Lind Hospital, Norwich.

Eastbourne Borough Sanatorium.—Miss Mather Fanny Thackray has been appointed sister. She was trained at Bethnal Green Infirmary, where she has since been sister.

Jessop Hospital for Women, Sheffield.—Miss Nina Whelpton Johnson has been appointed theatre sister. She was trained at Croydon General Hospital, and has since been staff nurse at the Plaistow Maternity Charity and District Nurses' Home, and sister at Southwark Infirmary, East Dulwich. She holds the certificate of the Central Midwives Board.

Malton, Norton, and District Cottage Hospital.—Miss Mabel Hartey has been appointed assistant nurse. She was trained at the Royal Infirmary, Halifax, and has since been staff nurse at the Hospital for Women, Soho Square, London. She has also done private nursing in Nottingham for three years.

Manchester Victoria Memorial Jewish Hospital. Miss Annie Elizabeth Jones has been appointed matron. She was trained at the Great Hospital, Dudley, and has since been nurse matron at the Tipton Isolation Hospital, and staff nurse at the Manchester Victoria Memorial Jewish Hospital. She has also done private nursing.

Queen Charlotte's Lying-in Hospital, London.—Miss Alice Mary Garratt has been appointed superintendent of the nurses' home. She was trained at Southwark Infirmary, East Dulwich, where she afterwards became sister. She has since been home sister at Bethnal Green Infirmary, and housekeeping sister at University College Hospital, London.

Queen Victoria's Jubilee Institute for Nurses.—Miss Katharine S. Macqueen has been appointed as an additional inspector. She was trained at the Royal Infirmary, Edinburgh, was appointed Queen's nurse in 1894 and was superintendent of the Cornwall County Nursing Association from July 1903 to January 1905.

St. Olave's Infirmary, Bermondsey, London.—Miss C. Dyne has been appointed charge nurse. She was trained at the Sunderland Poor Law Infirmary and the Sunderland Eye Infirmary, she has since been charge nurse at the Newcastle-on-Tyne Poor Law Infirmary and district nurse at Gateshead-on-Tyne.

St. George's Infirmary, Fulham Road, London.—Miss M. Adelaide Barker, Miss E. M. Page, and Miss R. White have been appointed sisters. Miss Barker was trained at Cleveland State Sick Asylum and has since done private nursing. Miss Page was trained at Greenwich Union Infirmary and has since been staff nurse and holiday sister in the same institution. Miss White was trained at St. Pancras Infirmary and has since been ward nurse at the Samaritan Free Hospital, London.

Shoreditch Infirmary.—Miss Emily Duncan has been appointed night superintendent, and Miss Elizabeth Mercer and Miss May McNicol ward sisters. Miss Duncan was trained at St. George's Hospital, London, and has since been charge nurse at Grove Hospital, Tooting, and sister and maternity sister at Lewisham Poor Law Infirmary. She holds the certificate of the Central Midwives Board. Miss Mercer was trained at Nottingham General Infirmary, and has since been charge nurse at Grove Hospital, Tooting, and pupil midwife at the Royal Maternity Hospital, Edinburgh. Miss McNicol was trained at the Western Infirmary, Glasgow, where she has since been staff nurse and ward sister. She has also been sister at Bellefield Sanatorium, Lanark, N.B.

West Cumberland Infirmary, Whitehaven.—Miss B. W. Stericker has been appointed sister. She was trained at St. Bartholomew's Hospital, London, and has since been sister at the Children's Hospital, Nottingham.

### To Nurses.

We invite contributions from any of our readers, and shall be glad to pay for "Notes on News from the Nursing World," "Incidents in a Nurse's Life," or for articles describing nursing experiences at home or abroad dealing with any nursing question from an original point of view, according to length. The minimum payment is 5s. Contributions on topical subjects are specially welcome. Notices of appointments, letters, entertainments, presentations, and deaths are not paid for, but we are always glad to receive them. All rejected manuscripts are returned in due course, and all payments for manuscripts used are made as early as possible after the beginning of each quarter.

### New Books for Nurses.

A Handbook of Nursing. Revised edition. For hospital and general use. (Philadelphia and London: J. B. Lippincott Company, 1905. Pp. 319. 28 illustrations. Price 5s.)

This is a new edition of a useful handbook of nursing, first published in 1878, when manuals of instruction for nurses were few and far between, and proportionately valued. Now, they flood the market, and the learner is often perplexed in making her choice of a text-book among so many that are good. It speaks well for the excellence of this work that it has stood the test of so many years, and yet thoroughly deserves its new setting. It is divided into three sections, the first dealing with the general principles of medical and surgical nursing, a chapter on the special nursing of children being included. The second is devoted to "Directions for Monthly Nursing," and the third to "Family Hygiene and Emergencies," in which common-sense treatment of everyday accidents is clearly detailed. The book concludes with a short list of abbreviations in common use in hospitals, also a vocabulary of some words whose meanings the nurse should know. The index is fairly complete, and the illustrations add to the inteerst and lucidity of the letterpress.

A Nurse's Handbook of Obstetrics. By Joseph Brow Cooke, M.D. Lecturer on Obstetrics to the New York City Training School for Nurses, etc. (Philadelphia and London: J. B. Lippincott Company, 1905. Second edition, revised. Pp. 403. 174 illustrations. Price 9s. net.)

Dr. Cooke's volume is by far the most comprehensive handbook on obstetrics for nurses that has yet been written, and although its price is somewhat prohibitive to many, it will amply repay its purchase to those able to buy a copy. Its frequent repetition of the same information adds unnecessarily to its bulk, but this error perhaps a third edition may remedy. On the other hand, the index is not full enough, nor always accurate in its page numbers. It is to be feared that a counsel of perfection is enjoined in the minute details of asepsis to be followed by the nurse in all her preparations for the arrival of the expected infant. In a wealthy house, and with plenty of help, they might be satisfactorily carried out, but it would be impossible to do so, single-handed, in the homes of the poor. The nurse's uniform, excellent in most respects, seems incomplete without the familiar bib to the apron; the detachable half sleeves are not altogether unknown in England. A new feature in works of this kind is the short chapter on "Maternal Impressions and the Control of Sex"—an ever-interesting question. The chapters dealing with the disorders of pregnancy are exceedingly good, especially the section on Eclampsia, fortunately, not a common complication in England at any rate. Another counsel of perfection is that a trained nurse should be in attendance during most of the pregnancy. This is usually impossible, though it would doubtless prevent many a complication arising either from the ignorance and folly of the patient and her friends, or from the unnatural stress and strain of modern civilised life.

Surgical Nursing. By Russell Howard, M.B., M.S. Lond., F.R.C.S.Eng. (London: Edwin Arnold 41 and 43 Maddock Street, Bond Street, W. 1905. Pp. 312, with 12 illustrations and 27 figures. Price 6s.)

This book presents in a concise, straightforward way the principles and methods of up-to-date surgery, with details of treatment in operation cases invaluable to the surgical nurse. The author says in the preface that "the active strides made by surgery in the past few years demands a great increase of knowledge on the part of the nurse, who must understand the principles on which the surgeon is working in order to render him efficient aid." A study of the book puts this knowledge within her reach. Many of the complicated subjects in nursing are dealt with clearly and simply, such as the aseptic process, its needs and details for observing and carrying out. The action of micro-organisms, specific infectious fevers, the presence of toxins in the blood, production and injection of anti-toxin, and its results. Several chapters are devoted to various operations, and details are given for preparation of the room, patients, and surgical appliances which will be needed; also, the after treatment of the patient and symptoms of complications. There are many figures and diagrams in the book, some of which are a great help in the study of the text, *i.e.*, diagram of the alimentary canal, and another of the main arteries of the body. Hæmorrhage and its arrest, burns, wounds, and fractures and their treatment are explained in different chapters. In that on fractures the names of splints (with figures) and manner of applying them are given. Details for nursing a case of fractured spine and a description of the spinal column have a chapter devoted to them. Besides being commendable for the use of nurses and medical students, there is much in it that the novice would find useful prior to her probation. All nurses will find the chapter on "infants" interesting and useful, especially the tables for feeding infants at various periods of life. Also the appendix, which gives ingredients for mouth-washes, eye-drops, enemata, etc., and a list of abbreviations used in prescriptions. The final chapter treats of elementary massage.

We have received from Mr. Henry Frowde, of the Oxford University Press Warehouse, a copy of "Catharine Grace Loch, Royal Red Cross, Senior Lady Superintendent of the Indian Army Nursing Service; a Memoir." The editor's name, Surgeon-Major Bradshaw, which appears in the advertisement of the book, is omitted from the title-page. Why?

### Christmas Novelties.

(By Our Shopping Correspondent.)

### THE AUSTRAL PURE WOOL CHEST AND LUNG PROTECTORS.

(The Nurses' Outfitting Association, Stockport.)

The Nurses' Outfitting Association at Stockport are now producing some very useful chest and lung protectors, which are highly recommended by the medical profession where regular heat has to be maintained. In cases of pneumonia these are invaluable, as they obviate any danger of chilling the patient when changing the poultices. Sleeveless jackets, recommended especially for pneumonia patients, can be procured for men, women, and children at prices varying from 1s. 6d. to 4s. 3d., according to size and quality; chest protectors range from 11d. to 2s. 6d., and the material, which is fine soft wool thoroughly carbonised and free from vegetable and other matter, can be obtained at 3s. 11d. and 5s. per yard.

### CHRISTMAS TOYS.

(Toler Brothers, Limited, Savoy Corner, Thames Embankment, Strand.)

Messrs. Toler are showing some delightful Christmas toys this year, including a box of ten assorted toys for very small people, which should appeal to the nursing world, as they are offered at 1s. per box carriage paid to any home or hospital. The Santa Claus hamper, too, containing six nice toys for boys and girls, an artistic celluloid Christmas card, ten of Raphael Tuck's Christmas cards, a big box of Christmas crackers, and a hanging calendar may be had for the modest sum of 1s. 9d. Comic Christmas marks are a speciality of the establishment, as are also some harmless and pretty drawing-fireworks which may safely be placed in the hands of children without danger to themselves or others.

### Notes and Queries.

**REGULATIONS.**

**The Editor is always willing to answer in this column, without any fee, all reasonable questions, as soon as possible.**

**But the following rules must be carefully observed.**

**1. Every communication must be accompanied by the name and address of the writer.**

**2. The question must always bear upon nursing, directly or indirectly.**

**If an answer is required by letter a fee of half-a-crown must be enclosed with the note containing the inquiry.**

*For Nurses Requiring Rest.*

(81) The East Grinstead District Nurse Committee have a furnished room which they would willingly offer to a nurse requiring rest, free of rent for three months. No attendance or board is given, so that it would not suit an invalid who could not wait upon herself. Miss Everard, Newlands, East Grinstead, Sussex, should be applied to for further particulars.

*New Zealand.*

(82) Can you tell me of any hospitals or nursing homes in New Zealand, and how a trained nurse going out might get on?—*Anxious Nurse.*

There are many good hospitals in New Zealand. Write to the matron of the Auckland Hospital, also to the matron of the Christchurch Hospital, for particulars, and they might also tell you about some nursing homes. We never recommend private nursing homes. If you do not join the staff of a hospital or a nursing home you should not go out there without good introductions and enough money to last you for twelve months if unemployed.

*Children's Nurse.*

(83) I have a great desire to become a children's hospital nurse. I have been a children's nurse for eight years. Will you tell me where to apply for training.—*E. F.*

You might write to the matron, North Eastern Hospital for Children, Hackney Road, Bethnal Green, E.; also to the Belgrave Hospital for Children, Kennington, S.E. If neither can receive you procure "How to Become a Nurse" from the Scientific Press, 28 and 29 Southampton Street, Strand. There you will find full details of the children's hospitals.

*Previous Experience.*

(84) Will you kindly tell me if three years' experience in Dr. Barnardo's Homes, sixteen months' fever training, and two years' private nursing would prevent my being taken on the staff of one of the largest general hospitals as a probationer for training three or four years? I am most anxious to have general training, but until now have been too young. *Hope.*

Not necessarily, although many matrons have an objection to previous training. See "How to Become a Nurse," published by the Scientific Press, Limited, then write to the matrons of the hospitals which seem most suitable to your case.

*West Africa.*

(85) I should be very glad if you could tell me to whom to apply for particulars of the West African nursing service.—*K. W.*

Write to the Hon. Secretary of the Colonial Nursing Association, Imperial Institute, London, S.W.

*Twelve Months' Training.*

(86) Where, please, can I get twelve months' training in medical and surgical nursing without paying a premium? *A Subscriber.*

You will find it very difficult to train for twelve months only, unless you go as a paying probationer, but you might find it worth while to advertise.

*Incurable Child.*

(87) Will you kindly tell me of a home into which I could get a little girl six years old? She has hip disease and has been sent out of hospital as incurable. Her mother drinks and is very unkind to her.—*Queen's Nurse.*

Write to the Home and Hospital for Incurable Children, Northcourt, Hampstead, or to the Children's Home, Coldash, Newbury, Berks.

**Handbooks for Nurses.**

| | Post Free. |
|---|---|
| "How to Become a Nurse: How and Where to Train." | 2s. 4d. |
| "Nursing: its Theory and Practice." (Lewis.) ... | 3s. 6d. |
| "Nurses' Pronouncing Dictionary of Medical Terms." | 2s. 6d. |
| "Complete Handbook of Midwifery." (Watson.) ... | 6s. 4d. |
| "Preparation for Operation in Private Houses." ... | 0s. 6d. |

Of all booksellers or of the Scientific Press, Limited, 28 & 29 Southampton Street, Strand, London, W.C.

### For Reading to the Sick.

CHRISTMASTIDE.

Night's silver veil o'er Bethlehem hung low:
The white flocks on the hill-slopes sleeping lay,
Dreaming of brooks and pastures far away.
A holy calm rested on all below,
Save that the fragrant wind swayed to and fro
The spreading olives. Wearied with the day,
The shepherds, too, had fall'n asleep. They say
The stars came down, grass, trees, and flowers did grow
Most eloquent in whisp'ring of the King;
Celestial choirs sang to the list'ning earth;
And angels came, a heav'nly host, to bring
To men glad tidings of that wondrous birth.
When o'er Judea's hills the gray dawn broke
The world in its Redeemer's smile awoke.

*H. R. Stark.*

Year by year He sets Himself before us, a little Child, in great humility, and bids us become like Him, that when He appears again, in His glorious Majesty, we may again be made like Him. Year by year, through His holy Nativity, He calleth us to behold Him, and crieth, by His very speechless Infancy, "Take My yoke upon you, and learn of Me; for I am meek and lowly in heart: and ye shall find rest unto your souls."

This is the special festival of humility, as of joy, a lowly joy, a joy of the lowly. Our Lord, from the manger, where for our sakes He deigned to lie, preaches to us humility. This was the beginning and end of His teaching. He taught it in action now, by His Birth; He taught it in all His Life and Sufferings; He not merely, as in the days of His Flesh, setteth before us, His disciples, a little child, and bids us become like it if we would "enter into the Kingdom of Heaven," He has Himself become that little Child.

Above all, treasure any season in which God Himself maketh thee lonely. When He brings thee back into thyself, seek not to go forth out of thyself. Whether it be by sickness, or by bereavement, or by any other sorrow, by want of the sympathy of the world, by distresses which make the heart sick and faint, go not forth out of thyself, but, with the prophet, stand in loneliness "upon thy watch, and set thee upon the tower;" dwell in Him, Who "is a most strong tower to all them that put their trust in Him."

How will all the longest trial shrink into a very nothing, when thy amazed soul shall enter into the brightness of His Eternal Light and Love!

*E. B. P.*

The Love of Him Who in a Manger lay,
Fulfil thee with the joy of Christmas-tide.
The Peace of God, that passeth not away,
Enfold thee; and the Comforter abide
To bless thee; till a touch shall end the fray,
And crown thee conqueror at thy Captain's side.

## Figures and Tables

**Figure f1:**
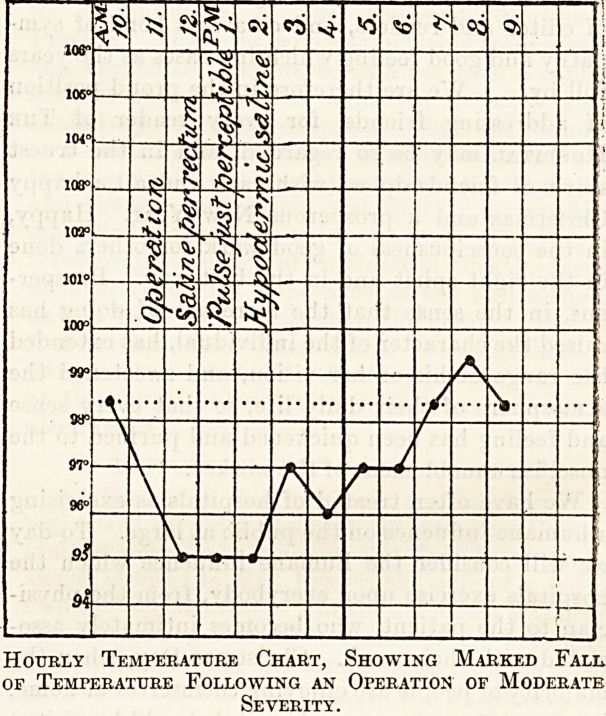


**Figure f2:**